# Women With Cerebral Infarction Feature Worse Clinical Profiles at Admission but Comparable Success to Men During Long-Term Inpatient Neurorehabilitation

**DOI:** 10.3389/fnagi.2021.663215

**Published:** 2021-11-18

**Authors:** Alexandra Kautzky-Willer, Jürgen Harreiter, Anita Thomas, Johannes Burger, Ulrich Schneeweiß, Carola Deischinger, Wolfhard Klein, Hermann Moser

**Affiliations:** ^1^Gender Medicine Institute, Gars am Kamp, Austria; ^2^Gender Medicine Unit, Division of Endocrinology and Metabolism, Department of Medicine III, Medical University of Vienna, Vienna, Austria; ^3^Neurologisches Therapiezentrum Gmundnerberg, Altmünster, Austria

**Keywords:** stroke, sex, gender, neurorehabilitation, Barthel index

## Abstract

**Objective:** Little is known about possible sex and gender differences in post-stroke neurorehabilitation outcomes. We aimed to analyze if functional performance, prevalence and impact of comorbidities at admission, and success of inpatient stroke-neurorehabilitation differ between men and women.

**Methods:** Retrospective cohort analysis of 1,437 men and 907 women with prior cerebral infarction treated at a neurorehabilitation clinic between 2012 and 2017; multiple linear regression was used to examine the influence of sex/gender as well as multiple confounders on health and functional outcomes. The main outcome measures were Barthel index (BI) at admission and its change during 4 weeks inpatient neurorehabilitation.

**Results:** Men had been diagnosed with osteoporosis less frequently than women but more often with type 2 diabetes mellitus, coronary artery or chronic kidney disease (*p* ≤ 0.01). Although twice as many women presented with pre-stroke depression compared to men, the risk of post-stroke depression detected during rehabilitation was comparable. Men were more likely to have less than 30 days between diagnosis and neurorehabilitation start than women (*p* < 0.03). At admission, women exhibited less autonomy, a lower BI, a higher pain score and worse 2-min walk test (2′WT) compared to men (*p* < 0.001). Among males osteoporosis and peripheral artery disease independently predicted BI at admission, in women it was pre-stroke depression, dementia, and arterial fibrillation. During neurorehabilitation, both sexes improved regarding BI, pain and walk tests (*p* < 0.001). Despite comparable rehabilitation effectiveness, women still had worse functional outcomes than males at discharge. Time after stroke to start of neurorehabilitation and length of the stay but, most strongly, the simple 2′WT at admission, and in women, pain intensity independently predicted post-stroke functional status and recovery.

**Conclusion:** Women presented with worse functional status at admission to neurorehabilitation. Although men and women showed similar rehabilitation effectiveness, women still displayed worse clinical outcome measures and higher levels of pain at discharge. Early access and gender-sensitive, personalized post-stroke care with more focus on different comorbidities and psychosocial factors like pain levels and management, could further improve neurorehabilitation outcomes.

## Introduction

Cerebrovascular disease is a major global burden causing 15% of deaths in Austria (Haast et al., 2012). Due to improved emergency medicine and admission to stroke units, the short term stroke case-fatality rates have decreased, however, at the same time, persisting impairments and disability have increased (Benjamin et al., 2019). Biological (sex), psychosocial and environmental (gender) differences may contribute to differences in stroke outcomes (Haast et al., 2012). However, the exact causes remain unclear and most studies investigated the acute care setting. Studies on stroke-neurorehabilitation are few in number, refer to outdated standards of care or include only small cohorts (Paolucci et al., 1998; van Meijeren-Pont et al., 2019). Despite inconsistent outcomes regarding the few analysis of differences between men and women (Di Carlo et al., 2003; Kelly-Hayes et al., 2003; Paolucci et al., 2006; Ones et al., 2009; Petrea et al., 2009; Pohl et al., 2013; Caglar et al., 2014; Ullberg et al., 2015; Willers et al., 2018), women are reported to have a worse rehabilitation success (Paolucci et al., 2006; Ullberg et al., 2015, 2016b) as well as a higher need for assistance and 3 fold higher admission rates to special care homes post-stroke in some studies (Petrea et al., 2009). Recent evidence shows that acute stroke care is comparable between both sexes in Austria regarding onset-to-door time, onset-to-needle time, rates of imaging or intravenous thrombolysis procedures (Gattringer et al., 2014). Therefore, despite equal quality of acute management, worse functional outcome of women may be ascribed to different comorbidities, pre-existing disabilities and more severe strokes (Arnao and Caso, 2014; Gattringer et al., 2014). Further, sex differences in access to or success of stroke-neurorehabilitation could contribute to differences in outcomes between men and women. Neurorehabilitation is well structured in Austria and available for every patient with health insurance, independent of income. However, the Austrian Acute Stroke Unit Registry reported that only 37% of the women and 41% of the men had neurorehabilitation within 3 months after hospital discharge and that the majority of patients lacked subsequent tracking. Therefore, little is known about rehabilitation success and long-term functional outcomes stratified by sex in Austria. On these grounds, we aimed to study physical and psychological comorbidities and functional outcomes of men and women with cerebral infarction at time of admission and discharge in an inpatient neurorehabilitation facility to identify potential discrepancies between men and women which might aid in improving stroke neurorehabilitation, and thus, long-term post-stroke outcomes in both sexes.

## Materials and Methods

This single-center cohort study was approved by the Local Research Ethics Committee (number K-84-15; Upper Austria, Linz). After exclusion of 407 patients with subarachnoid or intracerebral hemorrhage, 2,344 patients with cerebral infarction who were treated in a neurorehabilitation center in Austria from 2012 to 2017 were included in the analysis (Table 1). This institution is specialized in treatment of patients of Phase C [at least partially oriented, independent in some activities of daily living (ADLs) with participation in therapy for several hours daily] or D (patient is largely independent in ADLs, in need of some technical aid and professional supervision) (Austrian Society of Neurorehabilitation (OeGNR), 2018). In the neurorehabilitation center, an individualized training program to improve mental or motoric functions is implemented in guidance of a team of specialists. Several tests and questionnaires were performed to check for mental or physical disabilities and allow allocation of individual treatment programs involving speech or occupational therapists, physiotherapist, neuropsychologists, dietologists, and social workers. The assessment follows WHO International Classification of Functioning, Disability and Health (ICF) standards. Interventions are based on activities of daily life, are of high intensity and repetition rates and individualized according to the needs of every patient. Motivation and positive feedback further encourage the patient. The standardized duration of rehabilitation was 4 weeks. Data was obtained retrospectively from electronically stored data and from a review of the medical charts from the rehabilitation facility (full access). During the observation period, 2% of the patients left the neurorehabilitation program prematurely and were consequently lost to follow-up and excluded from this analysis. No patient died during neurorehabilitation.

**TABLE 1:** Baseline characteristics and comorbidities of men and women with diagnosis of cerebral infarction at admission to neurorehabilitation.

	Men	Women	*p*-value	Total
n (%)	1437 (61.31%)	907 (38.69%)		2344
**Age (years)**	**70.08 ± 11.75** (1437)	**72.1 ± 12.94** (907)	**<0.001**	70.86 ± 12.27 (2344)
**<65 years**	**444 (30.90%)**	**241 (26.57%)**	**<0.001**	685 (29.22%)
**65–79 years**	**689 (47.95%)**	**382 (42.12%)**		1071 (45.69%)
**80+ years**	**304 (21.16%)**	**284 (31.31%)**		588 (25.09%)
**Weight (kg)**	**83.98 ± 14.97** (1170)	**71.39 ± 14.82** (754)	**<0.001**	79.05 ± 16.13 (1924)
**Height (cm)**	**174.00 ± 7.76** (1040)	**161.23 ± 7.00** (671)	**<0.001**	168.99 ± 9.73 (1711)
BMI (kg/m^2^)	27.64 ± 4.35 (1033)	27.31 ± 5.19 (669)	0.171	27.51 ± 4.70 (1702)
BMI category*				
**Underweight (n)**	**4 (0.39%)**	**10 (1.50%)**	**<0.001**	14 (0.82%)
**Normal weight (n)**	**281 (27.28%)**	**228 (34.13%)**		509 (29.98%)
**Overweight (n)**	**494 (47.96%)**	**250 (37.43%)**		744 (43.82%)
**Obese (n)**	**251 (24.37%)**	**180 (26.95%)**		431 (25.38%)
Days between diagnosis and rehab start	318.54 ± 994.46 (1267)	332.33 ± 1026.21 (798)	0.762	323.87 ± 1006.62 (2065)
**Early access**	**301 (23.76%)**	**155 (19.42%)**	**0.021**	456 (22.08%)
<38 days (T1)	440 (34.73%)	257 (32.21%)	0.402	697 (33.75%)
38–84 days (T2)	407 (32.12%)	276 (34.59%)		683 (33.08%)
>84 days (T3)	420 (33.15%)	265 (32.21%)		685 (33.17%)
**Barthel index (BI)**	**87.61 ± 21.81** (1437)	**83.34 ± 23.65** (907)	**<0.001**	85.96 ± 22.63 (2344)
**Mean age adjusted BI** ^†^	**87.22**	**83.96**	**<0.001**	
**Autonomy status**				
**Poor ADL status (BI < 50)**	**128 (8.91%)**	**118 (13.01%)**	**<0.001**	246 (10.49%)
**Partial ADL status (BI 50–90)**	**333 (23.17%)**	**271 (29.88%)**		604 (25.77%)
**ADL autonomy (BI > 90)**	**976 (67.92%)**	**518 (57.11%)**		1494 (63.74%)
MMSE (Cognitive Function)	27.72 ± 3.59 (675)	27.78 ± 3.57 (450)	0.783	27.74 ± 3.58 (1125)
**VAS (pain intensity)**	**1.45 ± 2.38** (1164)	**1.86 ± 2.48** (745)	**<0.001**	1.61 ± 2.43 (1909)
**Pain**	**365 (31.36%)**	**307 (41.21%)**	**<0.001**	672 (35.20%)
**2 min walk test (m)**	**128.14 ± 48.64** (1063)	**114.15 ± 46.54** (615)	**<0.001**	123.01 ± 48.34 (1678)
**% of predicted normative reference value of 2**′**WT**^‡^	**73.81% ± 27.46%** (852)	**70.26% ± 27.88%** (517)	**0.021**	72.47% ± 27.66% (1369)
10 meter walk test (sec)	11.47 ± 13.85 (934)	12.43 ± 10.12 (569)	0.152	11.84 ± 12.58 (1503)
HADS-A score	6.23 ± 3.63 (146)	7.96 ± 11.34 (154)	0.079	7.12 ± 8.54 (300)
Anxiety	49 (33.56%)	67 (43.51%)	0.077	116 (38.67%)
HADS-D score	5.38 ± 3.88 (146)	5.11 ± 3.90 (154)	0.554	5.24 ± 3.88 (300)
Post-stroke depression	37 (25.34%)	34 (22.08%)	0.506	71 (23.67%)
**Pre-stroke depression**	**104 (7.24%)**	**130 (14.33%)**	**<0.001**	234 (9.98%)
**Pre- or post-stroke depression**	**136 (9.46%)**	**154 (16.98%)**	**<0.001**	290 (12.37%)
Dementia	74 (5.15%)	47 (5.18%)	0.973	121 (5.16%)
Hypertension	1155 (80.38%)	702 (77.40%)	0.083	1857 (79.22%)
**CAD**	**287 (19.97%)**	**115 (12.68%)**	**<0.001**	402 (17.15%)
Atrial fibrillation	338 (23.52%)	231 (25.47%)	0.284	569 (24.27%)
Heart failure	33 (2.30%)	23 (2.54%)	0.712	56 (2.39%)
Peripheral artery disease	8 (0.56%)	2 (0.22%)	0.224	10 (0.43%)
Patent foramen ovale	80 (5.57%)	45 (4.96%)	0.525	125 (5.33%)
**Diabetes mellitus Type 2**	**355 (24.70%)**	**130 (14.33%)**	**<0.001**	485 (20.69%)
Fatty liver	37 (2.57%)	21 (2.32%)	0.694	58 (2.47%)
**CKD**	**85 (5.92%)**	**32 (3.53%)**	**0.010**	117 (4.99%)
**Osteoporosis**	**35 (2.44%)**	**114 (12.57%)**	**<0.001**	149 (6.36%)
Number of comorbidities	1.80 ± 1.09 (1437)	1.76 ± 1.01 (907)	0.301	1.78 ± 1.09 (2344)
**Smoking**	**96 (6.68%)**	**42 (4.63%)**	**0.040**	138 (5.89%)

*Data are presented as means ± standard deviation (n) or number (column %). Bold font indicates statistical significance.*

*WHO classification;

*^†^ANCOVA adjusted; ^‡^Based on prediction equation by Bohannon et al. (2015); T1, minimum to lower tertile; T2, greater than lower tertile to upper tertile; T3, greater than upper tertile.*

We aimed to analyze possible sex differences in functional performance [Barthel index (BI) and functional assessment scores 2-min walk test (2′WT) and 10-meter walk test (10mWT)], prevalence and impact of comorbidities known to be associated with stroke risk and outcome [main diagnosis (ICD 10) patent foramen ovale, hypertension, atrial fibrillation, coronary artery disease (CAD), heart failure, diabetes mellitus, fatty liver, chronic kidney disease (CKD), peripheral artery disease (PAD), osteoporosis, dementia, depression (medical history, termed pre-stroke depression)], and success of inpatient stroke-neurorehabilitation. The primary parameter was BI at admission and the secondary parameter the change in BI following neurorehabilitation (ΔBI). Additional assessments during neurorehabilitation included evaluation of pain intensity (VAS), post-stroke depression (HADS-B > 8 score) and anxiety (HADS-A > 8 score), and Mini Mental State Examination (MMSE).

Furthermore, we wanted to explore whether early access to rehabilitation, which was defined as less than 30 days post-stroke, or the duration of rehabilitation were related to differences in neurorehabilitation outcomes between men and women.

The BI consists of a ten-item ordinal scale assessing performance in ADL and mobility (Mahoney and Barthel, 1965). Higher scores (0–100) reflect higher degrees of independence and ability to live at home. Effectiveness, reflecting the proportion of potential improvement achieved during rehabilitation, was calculated by this formula: (*BIatdischarge*−*BIatadmission*)÷(100−*BIatadmission*)×100 (Shah et al., 1990). Autonomy status at admission and discharge were defined as follows: poor autonomy for a BI < 50, partial autonomy for a BI from 50 to 90, and full autonomy for a BI > 90.

The Mini Mental State Examination (MMSE) consists of 30 items to assess cognitive function asking different questions with regards to orientation, attention, memory, language, and visual-spatial skills (Folstein et al., 1975). Higher scores (0–30) reflect higher cognitive function.

The Visual Analog Scale for Pain Assessment (VAS) is an unidimensional assessment tool of individual pain intensity using a line with a scale with numbers 0–10 with highest scores reflecting maximum pain and lowest numbers defining no pain at all (Haefeli and Elfering, 2006). The level of pain is marked by the patient on the line.

Pain status according to VAS was defined as follows: No pain for VAS = 0, Pain for VAS ≥ 1.

The Hospital Anxiety and Depression Scale (HADS) is a 14 itemed self-assessment tool to detect states of depression and anxiety (Zigmond and Snaith, 1983). Anxiety (HADS-A) and Depression (HADS-D) subscales consist of seven questions each with a scale from 0 to 3. Only patients who were thought to be at a higher risk for depression or anxiety took these tests at the rehabilitation facility.

The 2-min and 10-meter-walk-tests were performed to assess maximum walking distance within 2 min and walking time for 10 m and were performed in a quiet, uncarpeted corridor (Collen et al., 1990). Subjects were provided with clear instructions and were allowed to rest during the test periods, if required. Distance walked in 2 min was recorded in meters and time needed for 10 m was recorded in seconds.

In general, different age and sex norms have to be kept in mind in regard to walk tests, with men walking faster and farther than women (Bohannon et al., 2015). Therefore, comparisons in relation to reported normative reference values were included in the analysis (Bohannon et al., 2015).

“Sex” was used to indicate primarily biological differences and “gender” to describe predominant psychosocial (depression, anxiety, pain) and lifestyle-related influences (e.g., smoking, obesity) or time to neurorehabilitation. However, manifold interactions between biological and societal influences in clinical outcomes have to be considered.

The **statistical analysis** was performed using IBM SPSS Statistics Version 24. *P*-values < 0.05 were considered statistically significant. A descriptive analysis was performed on the entire dataset with missing data deleted pairwise. To compare independent groups of categorical variables, χ^2^ tests were performed. For comparisons of groups of continuous variables, a *T*-Test, One-Way ANOVA or Welch Test with Hochberg’s GT2 or Games-Howell *post hoc* tests, as appropriate, were performed. Additionally, sex differences in BI at admission were assessed using analysis of covariance (ANCOVA) adjusting for age. Differences between men and women concerning BI, VAS, and WTs at discharge were additionally assessed by ANCOVA adjusted for baseline (at admission). For a comparison of parameters at admission and at discharge, paired *T*-Tests were used. Correlations were assessed by Pearson correlation analysis, point-biserial correlation when one variable was dichotomous or Spearman correlation analysis when at least one variable was ordinal.

We performed two multiple linear regression models. First, we performed a cross-sectional analysis of how BI at admission depends on the covariates [sex, age, body mass index (BMI), days between diagnosis and rehab start, VAS, 2′WT, depression, dementia, hypertension, coronary artery disease, arterial fibrillation, heart failure, peripheral artery disease, patent foramen ovale, diabetes mellitus type 2, fatty liver, chronic kidney disease, osteoporosis, and smoking]. In a second, longitudinal analysis, we predicted ΔBI as a function of the covariates (all of the above plus the days in rehabilitation). Missing data of BMI, days between diagnosis and rehab start, VAS and 2′WT were imputed using multiple imputations. Thirty datasets were created and analyzed together by using fully conditional specification method.

## Results

### Clinical Characteristics at Admission

Men were significantly younger, taller, and heavier compared to women (Table 1). A significant relationship between sex and BMI category was observed.

Overall men displayed a better health status, reflected by a higher BI (men 88 ± 23; women 83 ± 24), 2′WT (men 128 ± 49; women 114 ± 47), and ADL autonomy than women (all: *p* < 0.001). Men were more likely to start rehabilitation within 30 days from diagnosis (early access), especially in the oldest age group (80+ years). However, overall, no sex difference was observed, neither in total days nor in those stratified in tertiles (<38, 38–84, and >84 days) of follow-up time, independent of age.

Moreover, male patients had less pain and pre-stroke depression. However, post-stroke depression was comparable in both sexes, affecting a quarter of the male patients tested. We observed significant differences between men and women in comorbidities: men had a lower prevalence of osteoporosis but suffered more often from CAD, diabetes mellitus and CKD compared to women (Table 1). Only in younger patients (<65 years) the number of comorbidities was higher in men (*p* < 0.02).

### Relationship of Barthel Index at Admission With Associated Variables

In the whole cohort, BI showed a positive **correlation** with the 2′WT (*r* = 0.612, *p* < 0.001), BMI (*r* = 0.115, *p* < 0.001), and MMSE (Supplementary Table 1). A negative correlation was found with the 10mWT (*r* = −0.409, *p* < 0.001), age, and number of comorbidities. BI was also associated with sex (*r*_pb_ = −0.092, *p* < 0.001), obesity, early access to rehabilitation, pre-stroke depression, dementia, hypertension, CAD, arterial fibrillation, heart failure, patent foramen ovale, CKD, osteoporosis, and smoking.

In subgroups stratified by sex, BI was related to obesity (*r*_pb_ = 0.106, *p* < 0.001) and CKD only in men, whereas BI was associated with heart failure (*r*_pb_ = −0.122, *p* < 0.001), CAD and osteoporosis in women (Supplementary Table 1).

A **multiple linear regression analysis** (*n* = 2344) with multiply imputed cases showed that among all independent factors only 2′WT (β = 0.697, *p* < 0.001), BMI (β = 0.116, *p* < 0.001), days between diagnosis and rehabilitation start (β = 0.043, *p* = 0.006), dementia (β = −0.042, *p* = 0.010) and PAD (β = 0.034, *p* = 0.028) significantly related with BI. The 2′WT proved to exert the strongest impact.

An analysis stratified by sex (men: *n* = 1437; women: *n* = 907) confirmed 2′WT (men: β = 0.695, *p* < 0.001; women: β = 0.690, *p* < 0.001) and BMI (men: β = 0.120, *p* < 0.001; women: β = 0.114, *p* < 0.001) to be significantly related to BI in both sexes. Amongst males, PAD (β = 0.056, *p* = 0.005), days between diagnosis and rehabilitation start (β = 0.055, *p* = 0.009), and osteoporosis (β = 0.040, *p* = 0.044) were significantly related to BI, in females it was depression (β = −0.068, *p* = 0.006), dementia (β = −0.058, *p* = 0.023), and arterial fibrillation (β = −0.055, *p* = 0.048).

### Clinical Re-evaluation at Discharge (After Neurorehabilitation)

Men still had a significantly higher BI at discharge (Men 90 ± 19; Women 86 ± 22; *p* < 0.001), lower prevalence of pain intensity (*p* < 0.001) and any pain and better WTs than women (Table 2). Both WTs remained significantly worse in females (*p* ≤ 0.006), even after adjustment for baseline values (*p* ≤ 0.03) or in relation to predicted reference values (*p* < 0.02).

**TABLE 2:** Stroke-related factors and functional outcomes of men and women at discharge.

	Men	Women	*p*-value	Total
Days in rehab	30.25 ± 5.97 (1437) [min: 21 / max: 56]	30.23 ± 5.74 (907) [min: 21 / max: 56]	0.952	30.24 ± 5.88 (2344)
**Barthel index (BI) at discharge**	**90.18 ± 19.11** (1437)	**86.07 ± 21.89** (906)	**<0.001** 0.076*	88.59 ± 20.33 (2343)
ΔBarthel index	2.57 ± 7.76 (1437)	2.71 ± 7.68 (906)	0.665	2.62 ± 7.73 (2343)
**Improvement of Barthel index**	**279 (19.42%)**	**223 (24.61%)**	**0.003**	502 (21.43%)
Effectiveness	23.15 ± 41.10 (578)	20.07 ± 43.33 (484)	0.236	21.74 ± 42.14 (1062)
**Autonomy status at discharge**				
**Poor ADL status (BI < 50)**	**95 (6.61%)**	**94 (10.38%)**	**<0.001**	189 (8.07%)
**Partial ADL status (BI 50–90)**	**299 (20.81%)**	**250 (27.59%)**		549 (23.43%)
**ADL autonomy (BI > 90)**	**1043 (72.58%)**	**562 (62.03%)**		1605 (68.50%)
**VAS at discharge**	**0.77 ± 1.62** (1140)	**1.03 ± 1.74** (725)	**0.001** 0.296*	0.28 ± 0.45 (1865)
**Pain at discharge**	**275 (24.12%)**	**239 (32.97%)**	**<0.001**	514 (27.56%)
ΔVAS	−0.63 ± 1.69 (1138)	−0.80 ± 1.86 (724)	0.056	−0.70 ± 1.76 (1862)
**2 min walk test at discharge (m)**	**149.70 ± 50.24** (931)	**133.79 ± 47.86** (551)	<0.001 0.011*	143.78 ± 49.94 (1482)
**% of predicted normative reference value of 2**′**WT^†^**	**86.74% ± 27.74%** (743)	**82.65% ± 27.76%** (459)	**0.013**	85.18 ± 27.80 (1202)
Δ2 min walk test	21.67 ± 29.59 (931)	19.53 ± 25.15 (551)	0.155	20.87 ± 28.03 (1482)
**10 meter walk test at discharge (sec)**	**8.89 ± 7.85** (803)	**10.28 ± 10.49** (497)	0.006 0.029*	9.42 ± 8.97 (1300)
Δ10 meter walk test	−2.64 ± 11.13 (803)	−2.42 ± 5.91 (497)	0.690	−2.56 ± 9.48 (1300)

*Data are presented as means ± standard deviation (n) or number (column %). Bold font indicates statistical significance.*

*p-values adjusted for baseline (at admission);

*^†^Based on prediction equation by Bohannon et al. (2015).*

No significant sex difference in neurorehabilitation effectiveness or ΔBI was observed. Slightly more women featured BI improvement. Accordingly, significant sex differences in BI at discharge vanished after adjustment for respective baseline values (Table 2). However, at discharge, men were still more likely to show autonomy in ADL, reflecting a significant relationship of sex and autonomy status (*p* < 0.001).

Following neurorehabilitation, BI, pain score and both WTs improved in the whole cohort as well as in the sex-segregated groups (all, *p* < 0.001). The same applied to the subgroup analysis stratified by time (in tertiles) between stroke and rehabilitation (all, *p* < 0.001). Rehabilitation effectiveness and ΔBI only differed between the first (<38 days) and the last tertile (>84 days) in both men (*p* = 0.002) and women (effectiveness: *p* = 0.008; ΔBI: *p* = 0.03), with higher effectiveness in the groups with earlier admission, but without sex differences in the three rehabilitation-take-up time subgroups.

Also, following subgroup stratification according to the respective autonomy status at admission, there was a significant improvement in the BI, the pain score and the WTs both in the entire cohorts as well as in the sex-disaggregated groups (all, *p* < 0.05). Only in the male subgroup with ADL autonomy at admission, no significant further improvement in BI could be observed. Additionally, greater effectiveness (*p* = 0.008) as well as ΔBI (*p* = 0.014) were observed in men compared to females in the patients with poor or partial autonomy status. On the contrary, in the subgroup with adequate autonomy, women had slightly greater ΔBI (*p* = 0.036).

### Associations With Change of Barthel Index

Change of Barthel index showed a positive **correlation** (Supplementary Table 1) with effectiveness, age, days in rehab, Δ2′WT, 10mWT, number of comorbidities, as well as a negative correlation with BI (admission and discharge). In addition, a negative correlation was observed in 2′WT (at admission: *r* = −0.293, *p* < 0.001) and Δ10mWT, BMI, MMSE, and pain at discharge. ΔBI was also associated with early access to rehabilitation (*r*_pb_ = 0.066, *p* = 0.003), obesity, CAD, arterial fibrillation, and patent foramen ovale.

An analysis stratified by sex showed that ΔBI was negatively related to BMI (*r* = −0.090; *p* = 0.004), MMSE and BI (admission and discharge) as well as with early access to rehabilitation (*r*_pb_ = 0.079, *p* = 0.005), obesity and patent foramen ovale in men. In addition, ΔBI was positively associated with 10mWT at discharge. In women, ΔBI correlated with number of comorbidities and was inversely related to BI at admission, pain intensity and arterial fibrillation. Therefore greater BI improvements related to lower BI at admission in both men and women.

A **multiple linear regression analysis** (*n* = 2343) with multiply imputed cases showed that only 2′WT (β = −0.242, *p* < 0.001), VAS (β = −0.055, *p* = 0.008), osteoporosis (β = −0.040, *p* = 0.045), and days in rehabilitation (β = 0.036, *p* < 0.001) significantly related with ΔBI among all included independent factors. If the analysis was restricted to the group with poor or partial ADL (*n* = 849), however, only days in rehabilitation (β = 0.242, *p* < 0.001) and VAS (β = −0.107, *p* = 0.003) remained significantly related to ΔBI. On the other hand, in the ADL autonomy group only (*n* = 1494), a significant relationship with diabetes (β = −0.097, *p* < 0.001) and osteoporosis (β = −0.052, p = 0.048) could finally be observed.

In the total cohort, a sex-segregated analysis (men: *n* = 1437; women: *n* = 906) confirmed 2′WT (men: β = −0.288, *p* < 0.001; women: β = −0.159, *p* < 0.001) and days in rehabilitation (men: β = 0.180, *p* < 0.001; women: β = 0.259, *p* < 0.001) to be significantly related with ΔBI in both sexes. Amongst female patients VAS (β = −0.089, *p* = 0.009) was also significantly related with ΔBI. In men, 2′WT proved to exert the strongest impact, in women it was days in rehabilitation.

A sex-specific analysis (men: *n* = 461; women: *n* = 388) of the subjects with poor or partial ADL only confirmed the significant relation with days in rehabilitation (men: β = 0.194, *p* < 0.001; women: β = 0.320, *p* < 0.001) in both sexes. Additionally, the relation with VAS (β = −0.115, *p* = 0.033) also remained significant in women.

Furthermore, sex-specific analysis of the ADL autonomous group (men: *n* = 976; women: *n* = 518) confirmed the significant relationship between diabetes (men: β = −0.080, *p* = 0.016; women: β = −0.125, *p* = 0.006) and ΔBI in both sexes, while in men only, the relationship with osteoporosis (β = −0.150, *p* < 0.001) was also verified.

## Discussion

In this study, 68% of both sexes underwent neurorehabilitation within the first 3 months and 87% of men and 85% of women within the first year post-stroke. No sex difference was seen in post-stroke rehabilitation take-up time after stratification of the period in tertiles although men and women starting within the first tertile after stroke had better rehabilitation outcomes than those in the third tertile. However, men (24 vs. 19%) more often had early access (<30 days), which was related to better functional status in our as well as in other studies (Wade et al., 1985; Paolucci et al., 1998). The difference in early access was especially observed in the very old subjects who had the worst BI. Therefore, the focus should be on early neurorehabilitation for all stroke patients and especially for women. Post-stroke loss to follow-up was reported from the national Austrian Acute Stroke Unit Registry as well as from Sweden with women being more likely to display ADL dependency, independent of stroke severity (Ullberg et al., 2016a; Willers et al., 2018). In another study, 22% of patients reported unmet rehabilitation needs at 12 months (Ullberg et al., 2016b). Patients more often suffered from post-stroke depression and insufficient pain management; female sex was a predictor of worse outcomes (Ullberg et al., 2016b). Application and timing of rehabilitation depends on the health care providers as well as on the ability and willingness of the patients, and probably, on psychosocial determinants (Ullberg et al., 2016a). However, post-stroke ADL scores were consistently worse in women even after adjustment for psychosocial factors like education, medical insurance, and marital status (Lisabeth et al., 2015).

At large, we confirm a higher stroke outcome severity reflected by higher degree of disabilities in women in the study at hand. Women more often showed a BI below 90, a poor autonomy status and worse 2′WTs. Within both sexes, the 2′WT was strongly related to BI and the strongest predictor for ΔBI. Also, after adjusting for baseline values – contrary to BI – the 2′WT remained significantly different between sexes and worse in females, even after comparison to sex-related normative values.

Better BI at admission was related to higher BMI in both sexes. This may be explained by the finding that malnutrition and sarcopenia are more harmful than overweight, which is associated with higher metabolic resources and better life expectancy in the elder (Flegal et al., 2013; Senoo and Lip, 2016; Oesch et al., 2017). The finding that BMI independently and significantly predicted BI in both sexes could also indicate greater benefit from neurorehabilitation in case of relatively higher lean/muscle mass. In another rehabilitation cohort, the lower mobility of women was explained by less muscle function limiting their physical recovery (Paolucci et al., 2006).

In the total group, as expected, PAD and dementia also predicted functional performance. Osteoporosis predicted neurorehabilitation recovery in all subjects with cerebral infarction but predicted post-stroke physical function at admission especially in the male subgroup. In addition, among the patients with satisfactory autonomy at admission, osteoporosis predicted ΔBI only in males, further supporting the strong impact of bone health on men’s health. This can also be seen in a recent study, which observed a negative relationship between bone mineral density and stroke especially in men (Zhu et al., 2021). On the other hand, the relationship between bone health and stroke risk has a stronger impact on women (Myint et al., 2014). Anyway, based on the association between muscle function and bone properties after stroke, osteoporosis is an important post-stroke complication (Yang et al., 2020). Post-stroke predisposition to instability and osteoporosis increase the risk of hip fractures which increase mortality risk in both sexes but particularly in men (Barcelo et al., 2021). Otherwise, females have much higher prevalence of osteoporosis in our as well as in other studies. The high osteoporosis prevalence of elder women needs special attention as only older women showed a 60% higher risk of fractures during post-ischemic stroke neurorehabilitation (Huang et al., 2017). Thus, altogether, regular post-stroke screening for osteoporosis and appropriate management is recommended in both men and women.

In this study, women were slightly older than men, which is in accordance with the observation that women have an increased incidence of stroke at an older age (Niewada et al., 2005; Kautzky-Willer et al., 2016; Renoux et al., 2017). In our study, women displayed less comorbidities than men only among younger patients. The number of comorbidities was negatively associated with BI at admission in the whole cohort but they did not independently relate to BI or predict its change. Particularly, very old women had a significantly worse autonomy status than their male counterparts. However, the fact that women had a worse BI remained significant after adjustment for age. Indeed, worse functional outcomes for women were reported independent of age and pre-stroke dependency (Phan et al., 2018).

In general, rehabilitation effectiveness did not differ between sexes; actually, slightly more women displayed any improvement of BI following neurorehabilitation. As females were discharged with an inferior autonomy status than men, women could benefit from earlier access and longer, more intense programs with special focus on functional outcomes, especially at a higher age. This hypothesis is supported by the finding that the days in rehabilitation strongly predicted functional recovery in both sexes but especially in women and in patients with poor or intermediate baseline autonomy status. Otherwise, time between diagnosis and rehabilitation had a significant impact on BI at admission in the total cohort and especially in men who had earlier access overall, and particularly, when older. Although a substantial number of subjects featured satisfactory autonomy at admission, we observed a significant improvement of all outcome parameters after 4 weeks neurorehabilitation except for ΔBI which only improved in women and not in men. As expected, recovery was greater in patients with poor and intermediate autonomy at admission in both sexes; however, males experienced greater neurorehabilitation success than women despite better BI at admission in this group. Therefore, neurorehabilitation is especially important for all patients with insufficient autonomy but appears to have a reasonably significant effect even in patients with adequate autonomy.

In women, greater pain scores impacted BI in a relevant manner. Moreover, pain independently predicted rehabilitation success in the overall cohort as well as in the female subgroup. Also, among patients with poor and intermediate autonomy status at admission, pain intensity independently predicted recovery in women only. Post-stroke pain is a common but underreported and undertreated phenomenon which is related to depression, cognitive and bodily dysfunction (Harrison and Field, 2015). Depression itself is an important stroke risk factor but may only play a minor role in affecting mobility status (Paolucci et al., 2006; Pan et al., 2011). In our analysis, pre-stroke depression was related to BI at admission in all groups and furthermore independently significantly impacted functional status in females only.

Post-stroke depression was comparable in both sexes in our analysis although its association with sex is controversial (De Ryck et al., 2014; Winstein et al., 2016). It prolongs motor recovery and associates with recurrence of stroke and mortality (Hackett and Pickles, 2014; Werheid, 2015; Guiraud et al., 2016). Worse quality of life in relation to mobility, pain and depression was shown in women up to 1 year post-stroke, even after adjusting for sociodemographic variables and stroke severity (Bushnell et al., 2014). In men, bodily dysfunctions could contribute to risk of post-stroke depression while in women pre- and post-stroke depression may contribute to greater pain and participation restriction (Phan et al., 2018).

We found that men more often presented with CAD, type 2 diabetes and CKD and were more likely to be smokers which is consistent with other large database (Kapral et al., 2005; Arnao and Caso, 2014). Some studies showed a stronger sex-dependent association of stroke-related disabilities with specific comorbidities (Kautzky-Willer et al., 2016; Marzona et al., 2018; Soriano-Reixach et al., 2018). Interestingly, arterial fibrillation predicted BI at admission only in the female subgroup. This is in accordance with other studies showing worse outcomes after stroke in females with arterial fibrillation including data from the Austrian Stroke registry (Steger et al., 2004; Cheng and Kong, 2016). In regard to rehabilitation recovery, diabetes emerged as independent predictor in all but especially in female patients with good baseline autonomy. Diabetes is an important stroke risk factor and is related to a high risk of disability because of diabetic complications and comorbidities, all with greater impact in women than men (Kautzky-Willer et al., 2016). A recent study evaluating the effect of chronic conditions on physical disability showed that while stroke led to higher steady physical disability after diagnosis, diabetes led to higher increments of physical disability in later stage disease (Chou et al., 2021). It appears that stroke trumps the effects of diabetic comorbidities on physical function in patients with poor autonomy, however, other chronic diseases, especially diabetes have a significant impact on patients with sufficient post-stroke autonomy.

### Study Limitations

A limitation of our study is the retrospective design, that we lack data on pre-stroke functional status, socioeconomic data and information on stroke severity, NIH stroke score or modified Rankin Scale score, and acute care and treatment. Furthermore women were slightly older. Age differences may impact disparities in mobility and recovery. However, age-adjusted analysis confirmed worse functional status and outcome in women. Gender-related intersection of social factors such as education, income or marital status may influence stroke outcome. Worse functional outcome of women might be ascribed to their worse social situation with more often being widowed, less caring relatives and a less advantageous socioeconomic situation. In future, intersecting social variables are important to be collected alongside biological variables and comorbidities in order to better understand their impact on long-term outcomes and to better identify the most vulnerable post-stroke populations (Di Carlo et al., 2003; Reeves et al., 2008; Eriksson et al., 2009).

A strength is the large number of Central European patients of a single neurorehabilitation center with standardized procedures reflecting a real-world rehabilitation scenario. Moreover, we also report some important psychosocial health determinants such as depression, anxiety and pain status which were, e.g., not available in the Austrian Acute Stroke Unit Registry (Arnao and Caso, 2014).

## Conclusion

Men and women with cerebral infarction differ in physical functions, comorbidities and early access to neurorehabilitation but experience comparable neurorehabilitation success. Women show more disabilities including worse ADL scores and poorer autonomy, at admission and discharge, particularly when older. In the total cohort, and especially in men, post-stroke time to rehabilitation start predicted functional performance at admission. The simple 2′WT most strongly predicted improvement of BI in men while it was the number of days in rehabilitation in women. Further, in women, pre-stroke depression predicted functional status at admission and pain intensity their functional recovery. Our results confirm the need of implementation of systematic comprehensive long-term stroke follow-up programs. More research is necessary to elucidate if modifiable variables like earlier access to and individualized length of neurorehabilitation, with focus on appropriate depression and pain management, can improve rehabilitation outcomes.

## Data Availability Statement

The datasets generated during analysis of this study are available from the corresponding author on reasonable request.

## Ethics Statement

The studies involving human participants were reviewed and approved by the Local Research Ethics Committee of Upper Austria, Linz. Written informed consent for participation was not required for this study in accordance with the national legislation and the institutional requirements.

## Author Contributions

AK-W wrote the manuscript. JH, JB, US, and HM contributed to study design and data collection. WK was responsible for data control/cleaning. AT performed the statistical analysis. CD and HM edited the manuscript. All authors reviewed and revised the manuscript critically for important intellectual content, approved the version to be published, and agreed to be accountable for all aspects of the work in ensuring that questions related to the accuracy or integrity of any part of the work are appropriately investigated and resolved.

## Conflict of Interest

The authors declare that the research was conducted in the absence of any commercial or financial relationships that could be construed as a potential conflict of interest.

## Publisher’s Note

All claims expressed in this article are solely those of the authors and do not necessarily represent those of their affiliated organizations, or those of the publisher, the editors and the reviewers. Any product that may be evaluated in this article, or claim that may be made by its manufacturer, is not guaranteed or endorsed by the publisher.

## Abbreviations

10mWT: 10-meter walk test
2 ′ WT: 2-min walk test
WT: walk test
ADL: activities of daily living
BI: Barthel index
BMI: body mass index
CAD: coronary artery disease
CKD: chronic kidney disease
MMSE: Mini Mental State Examination
PAD: peripheral artery disease
VAS: Visual Analog Scale for Pain Assessment
Δ BI: change of Barthel index.
