# Supplementary material for: Women With Cerebral Infarction Feature Worse Clinical Profiles at Admission but Comparable Success to Men During Long-Term Inpatient Neurorehabilitation
Source: Front Aging Neurosci. 2021 Nov 18;13:663215. doi: 10.3389/fnagi.2021.663215 (PMC8637730; doi:10.3389/fnagi.2021.663215)
Supplement: Supplementary file 1 [file Table_1.DOCX]

Supplementary Table 1**: Correlation analysis with Barthel index at admission as well as with the change of BI during rehabilitation (Δ BI).**

|  |  | **Men** | | **Women** | | **Total** | |
| --- | --- | --- | --- | --- | --- | --- | --- |
|  |  | BI at admission | Δ BI | BI at admission | Δ BI | BI at admission | Δ BI |
| Sex | r_pb_ |  |  |  |  | **-0.092** | 0.009 |
|  | p |  |  |  |  | **<0.001** | 0.665 |
|  | N |  |  |  |  | **2344** | 2343 |
| Age (years) | r | **-0.236** | **0.093** | **-0.322** | **0.106** | **-0.278** | **0.099** |
|  | p | **<0.001** | **<0.001** | **<0.001** | **0.001** | **<0.001** | **<0.001** |
|  | N | **1437** | **1437** | **907** | **906** | **2344** | **2343** |
| BMI (kg/m²) | r | **0.131** | **-0.090** | **0.090** | -0.003 | **0.115** | **-0.052** |
|  | p | **<0.001** | **0.004** | **0.019** | 0.945 | **<0.001** | **0.032** |
|  | N | **1033** | **1033** | **669** | 668 | **1702** | **1701** |
| Obesity | r_pb_ | **0.106** | **-0.058** | 0.064 | -0.019 | **0.086** | **-0.043** |
|  | p | **<0.001** | **0.028** | 0.054 | 0.559 | **<0.001** | **0.040** |
|  | N | **1437** | **1437** | 907 | 906 | **2344** | **2343** |
| Early access to rehabilitation (<30 days) | r_pb_ | 0.042 | **0.079** | 0.065 | 0.045 | **0.055** | **0.066** |
|  | p | 0.136 | **0.005** | 0.068 | 0.199 | **0.013** | **0.003** |
|  | N | 1267 | **1267** | 798 | 798 | **2065** | **2065** |
| Days in rehab | r | **-0.265** | **0.249** | **-0.240** | **0.289** | **-0.254** | **0.264** |
|  | p | **<0.001** | **<0.001** | **<0.001** | **<0.001** | **<0.001** | **<0.001** |
|  | N | **1437** | **1437** | **907** | **906** | **2344** | **2343** |
| BI at admission | r | 1 | **-0.504** | 1 | **-0.383** | 1 | **-0.454** |
|  | p |  | **<0.001** |  | **<0.001** |  | **<0.001** |
|  | N | 1437 | **1437** | 907 | **906** | 2344 | **2343** |
| BI at discharge | r | **0.937** | **-0.170** | **0.946** | -0.063 | **0.941** | **-0.125** |
|  | p | **<0.001** | **<0.001** | **<0.001** | 0.057 | **<0.001** | **<0.001** |
|  | N | **1437** | **1437** | **906** | 906 | **2343** | **2343** |
| Rehabilitation effectiveness | r | 0.062 | **0.624** | 0.110 | **0.642** | **0.084** | **0.631** |
|  | p | 0.135 | **<0.001** | 0.016 | **<0.001** | **0.006** | **<0.001** |
|  | N | 578 | **578** | 484 | **484** | **1062** | **1062** |
| MMSE | r | **0.439** | **-0.103** | **0.384** | -0.021 | **0.417** | **-0.072** |
|  | p | **<0.001** | **0.007** | **<0.001** | 0.655 | **<0.001** | **0.016** |
|  | N | **675** | **675** | **450** | 450 | **1125** | **1125** |
| VAS at admission | r | -0.012 | -0.005 | 0.007 | **-0.113** | -0.011 | -0.045 |
|  | p | 0.685 | 0.871 | 0.854 | **0.002** | 0.632 | 0.050 |
|  | N | 1164 | 1164 | 745 | **744** | 1909 | 1908 |
| Pain at admission | r_pb_ | 0.005 | 0.007 | 0.040 | **-0.129** | 0.011 | -0.044 |
|  | p | 0.864 | 0.807 | 0.271 | **<0.001** | 0.619 | 0.054 |
|  | N | 1164 | 1164 | 745 | **744** | 1909 | 1908 |
| VAS at discharge | r | -0.025 | -0.009 | -0.052 | **-0.153** | -0.042 | **-0.063** |
|  | p | 0.402 | 0.769 | 0.164 | **<0.001** | 0.069 | **0.007** |
|  | N | 1140 | 1140 | 725 | **724** | 1865 | **1864** |
| Pain at discharge | r_pb_ | -0.003 | -0.002 | -0.008 | **-0.129** | -0.013 | **-0.050** |
|  | p | 0.913 | 0.945 | 0.836 | **0.001** | 0.574 | **0.030** |
|  | N | 1140 | 1140 | 725 | **724** | 1865 | **1864** |
| Δ VAS | r | -0.018 | -0.005 | **-0.079** | 0.005 | -0.04 | -0.001 |
|  | p | 0.537 | 0.873 | **0.033** | 0.888 | 0.082 | 0.974 |
|  | N | 1138 | 1138 | **724** | 723 | 1862 | 1861 |
| 2 minute walk test ad admission (m) | r | **0.599** | **-0.324** | **0.625** | **-0.238** | **0.612** | **-0.293** |
|  | p | **<0.001** | **<0.001** | **<0.001** | **<0.001** | **<0.001** | **<0.001** |
|  | N | **1063** | **1063** | **615** | **614** | **1678** | **1677** |
| 2 minute walk test ad discharge (m) | r | **0.572** | **-0.250** | **0.604** | **-0.162** | **0.588** | **-0.219** |
|  | p | **<0.001** | **<0.001** | **<0.001** | **<0.001** | **<0.001** | **<0.001** |
|  | N | **931** | **931** | **551** | **550** | **1482** | **1481** |
| Δ 2 minute walk test | r | 0.038 | **0.081** | 0.036 | **0.125** | 0.041 | **0.094** |
|  | p | 0.241 | **0.013** | 0.398 | **0.003** | 0.118 | **<0.001** |
|  | N | 931 | **931** | 551 | **550** | 1482 | **1481** |
| 10 meter walk test at admission (sec) | r | **-0.406** | **0.179** | **-0.428** | **0.144** | **-0.409** | **0.167** |
|  | p | **<0.001** | **<0.001** | **<0.001** | **0.001** | **<0.001** | **<0.001** |
|  | N | **934** | **934** | **569** | **568** | **1503** | **1502** |
| 10 meter walk test ad discharge (sec) | r | **-0.515** | **0.127** | **-0.300** | 0.026 | **-0.416** | **0.082** |
|  | p | **<0.001** | **<0.001** | **<0.001** | 0.568 | **<0.001** | **0.003** |
|  | N | **803** | **803** | **497** | 496 | **1300** | **1299** |
| Δ 10 meter walk test | r | **0.153** | **-0.124** | **0.226** | **-0.217** | **0.163** | **-0.140** |
|  | p | **<0.001** | **<0.001** | **<0.001** | **<0.001** | **<0.001** | **<0.001** |
|  | N | **803** | **803** | **497** | **496** | **1300** | **1299** |
| Pre-stroke Depression | r_pb_ | **-0.088** | 0.032 | **-0.133** | 0.042 | **-0.118** | 0.037 |
|  | p | **0.001** | 0.223 | **<0.001** | 0.205 | **<0.001** | 0.073 |
|  | N | **1437** | 1437 | **907** | 906 | **2344** | 2343 |
| Pre- or Post-stroke Depression | r_pb_ | **-0.057** | 0.019 | **-0.091** | 0.024 | **-0.082** | 0.022 |
|  | p | **0.03** | 0.481 | **0.006** | 0.470 | **<0.001** | 0.293 |
|  | N | **1437** | 1437 | **907** | 906 | **2344** | 2343 |
| Dementia | r_pb_ | **-0.078** | 0.002 | **-0.097** | -0.013 | **-0.086** | -0.004 |
|  | p | **0.003** | 0.939 | **0.003** | 0.699 | **<0.001** | 0.858 |
|  | N | **1437** | 1437 | **907** | 906 | **2344** | 2343 |
| Hypertension | r_pb_ | **-0.069** | 0.009 | **-0.098** | 0.037 | **-0.077** | 0.020 |
|  | p | **0.009** | 0.738 | **0.003** | 0.264 | **<0.001** | 0.340 |
|  | N | **1437** | 1437 | **907** | 906 | **2344** | 2343 |
| Coronary artery disease | r_pb_ | -0.038 | 0.038 | **-0.101** | 0.055 | **-0.051** | 0.043 |
|  | p | 0.147 | 0.153 | **0.002** | 0.095 | **0.013** | 0.039 |
|  | N | 1437 | 1437 | **907** | 906 | **2344** | 2343 |
| Atrial fibrillation | r_pb_ | **-0.077** | 0.033 | **-0.207** | **0.094** | **-0.132** | **0.057** |
|  | p | **0.004** | 0.208 | **<0.001** | **0.005** | **<0.001** | **0.006** |
|  | N | **1437** | 1437 | **907** | **906** | **2344** | **2343** |
| Heart failure | r_pb_ | -0.025 | 0.030 | **-0.122** | 0.044 | **-0.066** | 0.035 |
|  | p | 0.349 | 0.254 | **<0.001** | 0.190 | **0.001** | 0.086 |
|  | N | 1437 | 1437 | **907** | 906 | **2344** | 2343 |
| Patent foramen ovale | r_pb_ | **0.091** | **-0.053** | **0.091** | -0.028 | **0.092** | **-0.044** |
|  | p | **0.001** | **0.045** | **0.006** | 0.404 | **<0.001** | **0.034** |
|  | N | **1437** | **1437** | **907** | 906 | **2344** | **2343** |
| Chronic kidney disease | r_pb_ | **-0.097** | 0.008 | -0.013 | 0.006 | **-0.059** | 0.005 |
|  | p | **<0.001** | 0.762 | 0.691 | 0.849 | **0.004** | 0.816 |
|  | N | **1437** | 1437 | 907 | 906 | **2344** | 2343 |
| Osteoporosis | r_pb_ | 0.000 | -0.032 | **-0.084** | 0.000 | **-0.065** | -0.010 |
|  | p | 0.992 | 0.226 | **0.012** | 0.989 | **0.002** | 0.616 |
|  | N | 1437 | 1437 | **907** | 906 | **2344** | 2343 |
| Number of comorbidities | r | **-0.104** | 0.032 | **-0.245** | **0.086** | **-0.159** | **0.053** |
|  | p | **<0.001** | 0.225 | **<0.001** | **0.010** | **<0.001** | **0.011** |
|  | N | **1437** | 1437 | **907** | **906** | **2344** | **2343** |
| Smoking | r_pb_ | 0.034 | -0.015 | 0.064 | -0.057 | **0.048** | -0.030 |
|  | p | 0.200 | 0.572 | 0.053 | 0.084 | **0.019** | 0.149 |
|  | N | 1437 | 1437 | 907 | 906 | **2344** | 2343 |

r Pearson’s product moment correlation coefficient, ρ Spearman's rank correlation coefficient, r_pb_ point-biserial correlation coefficient. Bold font indicates statistical significance.
